# Old Drug, New Pain. Roles and Challenges of Methadone Therapy in Pediatric Palliative Care: A Systematic Review

**DOI:** 10.3389/fped.2022.874529

**Published:** 2022-05-27

**Authors:** Francesca Benedetti, Silvia Zoletto, Annalisa Salerno, Irene Avagnina, Franca Benini

**Affiliations:** ^1^Pediatric Residency Program, University of Padova, Padova, Italy; ^2^Pediatric Pain and Palliative Care Service, Department of Women's and Children's Health, University Hospital of Padova, Padova, Italy

**Keywords:** children, pain, methadone, life limiting disease, opioids, pediatric palliative care (PPC)

## Abstract

**Background:**

Pediatric palliative care (PPC) is defined as the prevention and relief from suffering of families and children with life-limiting (LLDs) or life-threatening diseases (LTDs). These patients often experience pain, with morphine being the most widely used drug to treat it. Few studies investigated the role of methadone in PPC patients, although it is considered among the most effective and underutilized drugs in PPC.

**Objectives:**

Our aim was to evaluate the efficacy, safety, and dosage of methadone in PPC.

**Methods:**

Between August and October 2021 PubMed, Scopus and the Cochrane Library were searched for studies on the use of methadone in children with LLDs and LTDs. Articles were included if they met the following criteria: published in the last 10 years, English language, patients aged 0–23 years; children enrolled in a PPC center or receiving declared support from a PPC service; reporting of specific data on methadone in interventional trials, observational studies, or case series on >10 patients. The reporting of the article was guided by the PRISMA guidelines, and a critical appraisal of the included studies was performed using the JBI-tool.

**Results:**

After duplicates removal and full-text assessment, four studies were included and another one was added after checking the references of the retrieved papers. All were retrospective, and the literature is concordant in documenting the lack of evidence. A total of 116 children received methadone in PPC. From our review emerges the poor quality of data collection: in only one study pain was assessed with standardized scales. All studies documented the effectiveness of methadone in treating complex pain, either nociceptive or neuropathic. No serious adverse events were reported, with no cases of cardiac arrhythmias.

**Conclusion:**

Our results suggest that methadone could represent a suitable strategy for treating pain in PPC. However, the evidence base is insufficient, and further research is warranted.

## Introduction

Pediatric palliative care (PPC) is defined as the prevention and relief from suffering of children with life-limiting or life-threatening diseases and their families (1). Pain is one of the most frequent and severe symptoms experienced by children in PPC (2). This symptom is often not well-managed, especially at the end-of-life period (3).

Opioids play a central role in the management of pain in PPC, with morphine being the most widely used drug (4, 5). Among other opioids, methadone could be a suitable option to treat moderate-to-severe pain. It is a synthetic opioid endowed with some properties that are useful in treating opioid-tolerant and selected opioid-naïve pediatric patients (6). First, methadone is not only a μ and δ opioid receptors agonist, but also antagonizes NMDA receptors and inhibits the re-uptake of serotonin and noradrenaline, therefore acting against both nociceptive and neuropathic pain (4, 7, 8). With respect to other opioids, it offers peculiar pharmacological characteristics, including the availability of a liquid oral formulation, lack of active metabolites, intestinal elimination, and long half-life (7, 9). It is also associated with a low cost for the healthcare system (10). Despite these potential advantages, only few studies have addressed the use of methadone in PPC, especially in the first-line setting (8, 11). To our knowledge, a systematic review on the use of methadone in PPC has not been conducted to date. Furthermore, there is still uncertainty about the most suitable equianalgesic doses of methadone.

In this systematic review, we evaluated the efficacy, safety, and dosage of methadone in PPC.

## Methods

### Selection Criteria

A preliminary research was conducted in August 2021 to rule out the possibility of an already existing review on the topic and to identify the keywords and selection criteria. Articles published in the last 10 years in English language were considered for inclusion if they met the following criteria: patients aged 0–23 years (12, 13); children must be enrolled in a PPC center or were receiving a declared support from a PPC service (14); reporting of specific data on methadone therapy in interventional trials, observational studies or case series on >10 patients.

### Search Criteria

A systematic review of literature was performed following the 2009 Preferred Reporting Items for Systematic Reviews and Meta-Analysis (PRISMA) guidelines (15), and the Joanna Briggs Institute (JBI) critical appraisal checklist for case series (16). Two authors independently applied the JBI tool for the risk bias evaluation to the selected articles; a third author was involved in case of disagreement.

Three databases (PubMed, Cochrane Library and Scopus) were browsed until October 30th, 2021. Searches used both controlled MeSH and free terms, and the descriptors were combined using Boolean operators. The search strategy and the key terms were adapted to the different databases (e.g., “methadone,” “pain,” “child/infant/adolescent,” “pediatric,” “palliative care,” “pediatric palliative care,” “end of life,” “life limiting diseases”).

Titles and abstracts of the retrieved articles were independently assessed by three Authors (FB, AS, and SZ) to identify the papers specifically referring to PPC; then, the full text of each paper was reviewed to assess the other inclusion criteria. The references of identified articles were screened to identify additional publications of particular relevance, even when not meeting all the selection criteria. Any disagreement was resolved by involving a fourth assessor (IA). Discarded papers could be considered for the Discussion on methadone therapy.

We designed a data extraction form, which two authors used to extract data from selected studies. Extracted data were cross-checked. Disagreements were settled by discussion and, if necessary, with involvement of a third researcher.

The extraction form included: name of first author, publication date, study design, study aim, sample size, setting, methadone treatment length, posology, method of administration, concurrent medications, efficacy, and adverse events. We sought data about efficacy/safety of methadone and its dosing. Any measure of pain was eligible for efficacy evaluation (if Self-measurement was not available, parental assessments were also considered valid). We did not prepare a protocol and the review was not registered.

## Results

**Figure 1:**
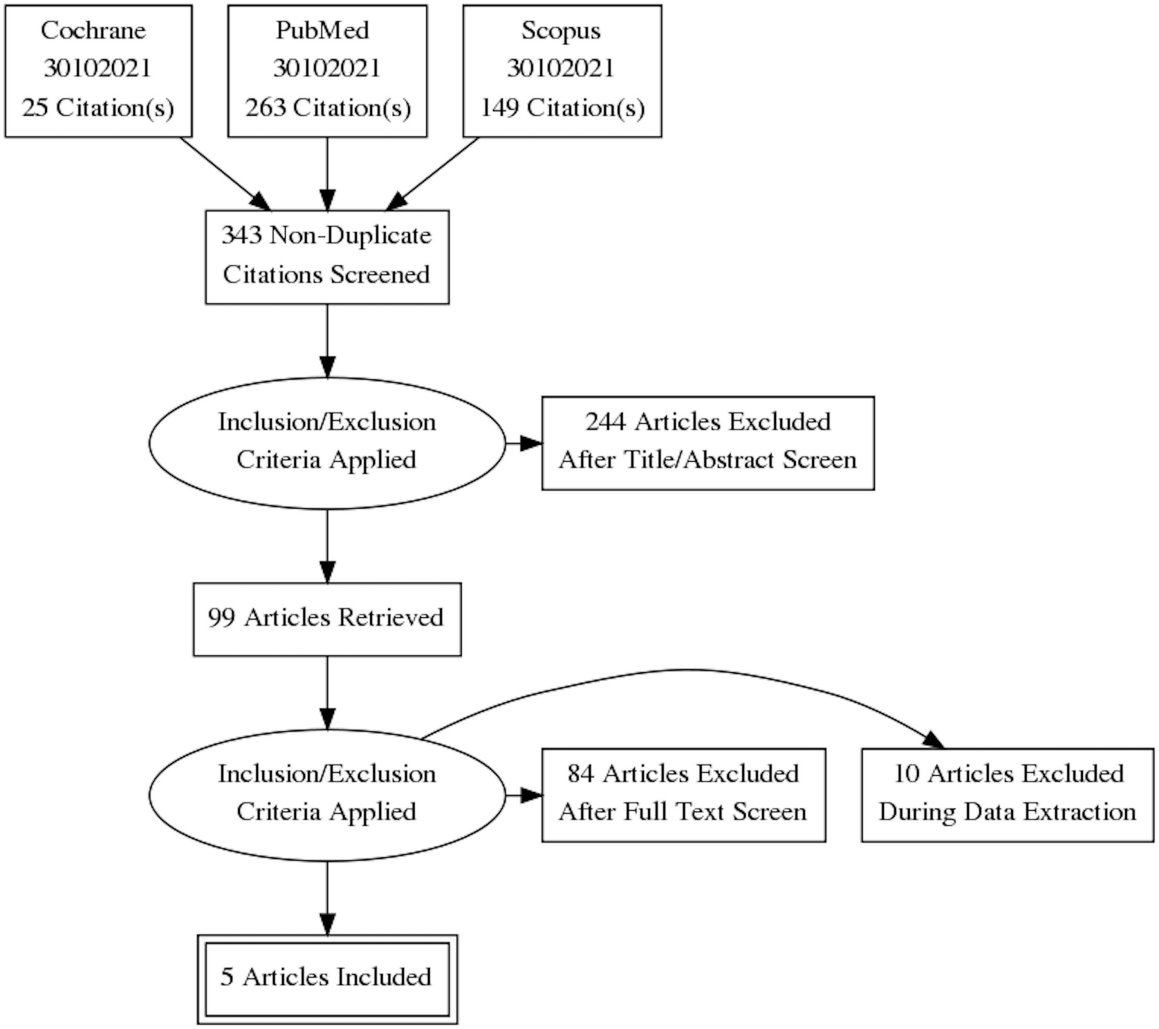
PRISMA flow diagram for study inclusion.

The initial search identified 437 records; after duplicates removal, 343 articles were screened. Of them, 99 articles underwent full text evaluation for eligibility and 4 papers (10, 17–19) met the inclusion criteria (Figure 1). Another paper (20), published >10 years ago, was identified after checking the references of the retrieved papers. Hence, a total of 5 studies were considered for the present systematic review (10, 17–20).

Table 1 summarizes the key characteristics of the identified studies. No interventional studies were found. All identified studies were retrospective.

**Table 1:** Studies included in the review.

**References**	**Study design**	**Aims (treatment of refractory pain)**	**Number of patients, setting**	**Other drugs**	**Median or *mean* duration (range)**	**Median or *mean* mg/kg TDD (range)**	**Time per day**	**Efficacy findings**	**Adverse events**
Madden et al. (10)	Retrospective, monocentric	Efficacy and safety of methadone as first-line long-acting opioid for analgesia treatment/refractory pain in children with advanced cancer	52, palliative care	No other drugs mentioned	17 (IQR, 9–26) days from BL to F1; 55 (IQR, 35–64) days from BL to F2	To start: 0.1 (IQR 0.1–0.1)	2 PO	Significant improved in pain, insomnia, and fatigue	None
Hall et al. (17)	Retrospective, monocentric	Efficacy and safety of methadone as adjunctive therapy for refractory pain in children with advanced cancer	20, inpatient, all patients in the last 4 weeks of life (end-of life care)	Yes (anti-anxiety, antidepressants, antipsychotics, sedation therapy, gabapentinoids, opioids, steroids)	32 days (2–323)	(0.09–7.76)	–	Valuable adjunctive therapy for nociceptive and neuropathic pain and to prevent opioid hyperalgesia and tolerance	No relevant AEs reported
Davies et al. (20)	Retrospective, monocentric	Effects of switching from high-potency opioids to methadone in treatment of severe pain in pediatric advanced cancer	17, 6 started methadone as outpatients	No other analgesic drugs were reported, but self-administred treatments are mentioned	36 days (1–199)	–	3 PO; intravenous: 24-h infusion	Subjective improvement in pain control was reported in 16/17 patients (94%)	One patient experience mild AEs
Mott et al. (18)	Retrospective, bicentric	Efficacy, dosing, and side effect of methadone switching in children with oncological (75%) and non-oncological life-limiting diseases	16, seven received methadone in a palliative care context and four at home	Yes (alternate analgesic medications, antibiotics, anti-epileptic drugs, anti-emetics, steroids, target treatment for underlying diseases)	–	Three patients began as adjuvant at initial dose of 1 mg/day. One naïve patient started with a pro kg dose	3 or 4 PO, SL or NG. Intravenous or subcutaneous: 24-h infusion	15 patients (94%) did not interrupt methadone and experienced improved pain control or reduced requirement of breakthrough medications. One patient interrupted it to subjective lack of effect	Drowsiness, unsteadiness, itch, constipation, transient bradypnoea and behavioral behavioral change as most common adverse events. Abstinence syndrome symptoms (*n* = 3), methadone reduction for AEs (*n* = 2).
Palat et al. (19)	Retrospective, monocentric	Efficacy and safety of methadone as adjunctive or single treatment for pain in children with advanced cancer	11, hospital setting (8), home (3)	Yes (morphine, fentanyl, tramadol, paracetamol, NSAIDs, amitriptyline, valproate, and gabapentin)	50 (7–307) days		1, 2 or 3 PO	Adequate analgesia in five patients, uncontrolled in 1, unchanged pain in 3. Not valuable in two patients	Nausea (3; 27%) and tachycardia (1; 9%).

*TDD, total daily dose. IQR, interquartile range. BL, baseline. F1, follow-up 1. F2, follow-up 2. AEs, adverse events. PO, per os. SL, sublingual. NG, nasogastric. NSAIDs, nonsteroidal anti-inflammatory drugs*.

Table 2 summarizes the quality determination of the studies included assessed with the JBI critical appraisal checklist for case series. Main bias observed in the studies was measurement of outcome, with pain monitoring being performed with a validated tool only in two studies. On the other hand, while describing methadone use, four studies correctly reported dosage and morphine equivalent dose. One study presented a bias related to missing data. Other bias were related to result reporting, with two studies in which methadone use is not contextualized to the patient's general clinical picture and one study in which outcomes were difficult to assess. Moreover, in two studies statistical analysis was intentionally not performed.

**Table 2:** Critical appraisal of included studies based on JBI I Critical Appraisal Checklist for Case series.

**textbfAuthors**	**Q1**	**Q2**	**Q3**	**Q4**	**Q5**	**Q6**	**Q7**	**Q8**	**Q9**	**Q10**
Davies et al. (20)	Y	Y	N	Y	Y	Y	N	Y	Y	NA
Hall et al. (17)	Y	Y	N	Y	Y	Y	Y	N	Y	Y
Madden et al. (10)	Y	Y	Y	Y	Y	Y	N	Y	Y	Y
Mott et al. (18)	Y	Y	N	Y	Y	Y	Y	Y	Y	Y
Palat et al. (19)	Y	Y	Y	Y	Y	Y	Y	N	Y	NA

*Y, yes; N, no; U, unclear; NA, not applicable. Q1Were there clear criteria for inclusion in the case series? Q2Was the condition measured in a standard, reliable way for all participants included in the case series? Q3Were valid methods used for identification of the condition for all participants included in the case series? Q4Did the case series have consecutive inclusion of participants? Q5Did the case series have complete inclusion of participants? Q6Was there clear reporting of the demographics of the participants in the study? Q7Was there clear reporting of clinical information of the participants? Q8Were the outcomes or follow up results of cases clearly reported? Q9Was there clear reporting of the presenting site(s)/clinic(s) demographic information? Q10Was statistical analysis appropriate?*

In the selected studies, a total of 116 pediatric patients received methadone; among them, 112 (96%) were affected by cancer pain.

The main findings of the identified studies are summarized below. Studies are listed according to the number of patients evaluated.

### Efficacy and Safety

In a monocentric retrospective study, Madden et al. (10) evaluated methadone as a first-line therapy in 52 children [median age 12.5 years (interquartile range 6.8–16.3)] with advanced cancer and uncontrolled pain, either opioid-naïve (*n* = 2; 4%) or receiving only immediate-release opioid (*n* = 50; 96%). Efficacy was assessed through an *ad hoc* pediatric symptom assessment system evaluating 11 physical and psychological symptoms. A five-point Likert scale (score 0–4) was used to register child and parent response for each symptom. Pain improved significantly over the entire follow-up period, as scored by the child (from a mean of 3.59 ± 0.61 standard deviation (SD) points to a mean of 1.80 ± 1.13 at the first follow-up) and the parent (from a mean of 3.46 ± 0.66 points to a mean of 1.35 ± 1.25 at the first follow-up). A similar trend was observed for insomnia and, partially, for fatigue. No cardiac arrhythmias, in particular no prolongation of QTc, or opioid-associated neurotoxicity (somnolence, hallucination, or myoclonus) were reported.

In a single-center, retrospective study, Hall et al. (17) evaluated 20 hospitalized children [mean age 16 years (range 2–24)] who were receiving methadone as adjunctive therapy to treat nociceptive (*n* = 18; 90%) or neuropathic (*n* = 2; 10%) advanced cancer pain during the last 4 weeks of life. Methadone was continued in 15 patients (75%) during the entire follow-up. Of the 20 patients, 8 (40%) received an ECG before starting methadone, and 6 had a monitoring of the ECG after initiation of therapy; no patient discontinued methadone due to an increased QTc interval. No other adverse events were mentioned.

In a retrospective, monocentric study on 17 children with advanced cancer [median age 8.9 years (range 2–18)], Davies et al. (20) assessed the effects of switching from high-potency opioids to methadone. Six of the 17 patients (35%) started methadone as outpatients, and only one required readmission to the hospital. A subjective improvement in pain control was achieved in 16/17 patients (94%), who remained on methadone until death, for a median time on treatment of 36 days (range from 1 to 199 days). Seventeen patients (94%) remained on methadone until death and 1 (6%) interrupted due to low efficacy and mild adverse events (somnolence, possible hallucinations, and mild respiratory depression).

In a bicentric retrospective study (18), 16 children with a life-limiting disease (cancer, *n* = 12; neurological conditions, *n* = 3; cardiac diagnosis, *n* = 1) received methadone for incomplete analgesia on other high-potency opioids (*n* = 9), adverse events from other opioids (*n* = 6) and desire to use oral analgesic (*n* = 3). Their mean age was 7.4 (1.3–16) years. Seven patients received methadone in a palliative care specialized setting and four were treated at home. Fifteen patients (94%) did not interrupt methadone and experienced improved pain control or reduced requirement of breakthrough medications. The most common symptoms reported in the early phase of the treatment were drowsiness, unsteadiness, itch, constipation, transient bradypnea and behavioral change. One patient had a treatment-emergent seizure with methadone being among the potential drugs involved. During methadone withdrawal, three patients experienced abstinence syndrome and pain rebound. In two patients, dose reduction was necessary due to adverse events.

In a monocentric retrospective study conducted in a low-resource setting in India, Palat et al. (19) evaluated methadone treatment in 11 children [median age 12 years (range 4–16)] with advanced cancer who were suffering from neuropathic (*n* = 3; 27%) or mixed (*n* = 8; 73%) pain and for whom first-line opioid therapy was ineffective (*n* = 10, 91%) or associated with adverse events (*n* = 1, 9%). Methadone was started in a hospital setting (*n* = 8) or at home (*n* = 3). Nine children received more than one administration of methadone (median duration 50 days, range 7–307). For three children, methadone was administered as the sole analgesic treatment. Five of the nine children who received more than one dose of methadone achieved adequate analgesia. The only adverse events reported were nausea (3/11; 27%) and tachycardia (1/11; 9%).

### Dosing and Equianalgesic Conversion

In the retrospective study of Madden et al. (10) the median initial dose of methadone was 0.1 mg/kg PO twice daily. Dosage adjustments could be performed as necessary. In total, 33 patients (70%) at first follow-up evaluation and 23 (79%) at second follow-up did not need a change in dose of methadone. In the study by Hall et al. (17) methadone doses ranged from 0.09 to 7.76 mg/kg daily. The route of administration varied during the follow-up period: five patients (25%) received only oral therapy, 7 (35%) i.v. intermittent therapy, 6 (30%) oral and i.v. intermittent therapy, 1 (5%) continuous infusion therapy and 1 (5%) oral, i.v. intermittent and continuous infusion.

Both Davies et al. (20) and Mott et al. (18) reported data on methadone use in patients previously exposed to other high-potency opioid medications. In these cases, the strategies used for opioids to methadone conversion were specifically analyzed. In the study by Davies et al. (20), 10 patients (59%) had a rapid conversion to methadone; the other patients progressively reduced the previous opioid over a few days while methadone was increased. The calculated total daily methadone dose (TMDD) was then divided into three equal daily doses. A breakthrough dose of 10% of the TMDD could be administered every 2 h, if necessary. Conversion ratios varied between 60:1 and 1:2. The highest ratio was reported in a patient previously treated with the highest dose of morphine equivalent daily dose (MEDD) for the longest time (600 mg for 217 days) reported in the study. On the other hand, the 1:2 conversion ratio was documented in the patient with the poorest control of pain. i.v. methadone was prescribed to 6 (35%) patients using an equianalgesic dose of 80% of TMDD except in cases of low pain control, when a 100% of the TMDD was administered. This i.v. dose was given as a 24-h infusion and breakthrough doses varied between 1/3 and 1-fold the hourly dose given as patient/parent-controlled analgesia.

In the study by Mott et al. (18), one patient started methadone as a first-line therapy and the dose was calculated pro kg. Three patients began methadone as adjuvant (initial dose 1 mg) therapy and moved to methadone monotherapy over a few days/weeks, and 12 patients rapidly switched from high-dose opioids to methadone. In this last group of patients, conversion ratios varied between 2:1 and 150:1; however, conversion ratios between 10:1 and 20:1 were the most frequently applied (50% of patients). A greater ratio was applied to patients with higher previous exposure to opioids. Indeed, a 150:1 conversion ratio was used in the patient who had received the highest dose of previous MEDD (10,800 mg), while 2:1 equianalgesic dose was applied to the patient with the lowest MEDD (12 mg).

Routes of administration were enteral (*n* = 8, 50%), sublingual (*n* = 2, 12.5%), intravenous (*n* = 4, 25%) and subcutaneous (*n* = 2, 12.5%). When given enterally, methadone was divided into three or four daily doses, while i.v. and s.c. formulations were given as a 24-h infusion.

In the study by Palat et al. (19), the starting dose was calculated on previous patient MEDD and was divided into one (six children), two (one child), or three (four children) daily administrations. All children received oral methadone: mainly the liquid formulation, but two of the 11 children received tablets.

## Discussion

Methadone presents several characteristics that could suggest its suitability in PPC: multiples routes of administration (oral, rectal, nasal, sublingual, subcutaneous, epidural or intravenous) including liquid formulation (21), safe use in renal failure, good oral bioavailability (80–90%), intestinal elimination and long half-life that allows fewer administrations per day (7). Moreover, methadone has a low cost (22, 23) making it a feasible option also in low-income countries (19). However, methadone also presents some issues, such as the high variability in individual response to treatment. This can be due to its widely varying plasmatic half-life (8–90 h) which may lead to accumulation (7, 24, 25). Furthermore, methadone has a high lipophilicity and therefore fat tissue can act as a drug reservoir, making plasma concentration unpredictable (25), and the equianalgesic conversion from other opioids remains unclear. Therefore, methadone is underused in PPC, and only few experiences have been published.

Indeed, in our systematic review, we were able to identify only five studies on methadone patients receiving PPC. Remarkably, all studies were uncontrolled and retrospective, and current literature is concordant in documenting the lack of evidence on this topic (8, 26–28).

It must be pointed out, however, that interventional studies are not always feasible in the PPC setting and therefore anecdotal observations can have a role in providing evidence on drug therapy in this population (29).

In the identified studies, the total population of children who received methadone in PPC was overall limited (116 patients), and the wide majority of them was affected with cancer-related pain. However, oncological patients represent only a minor proportion of children needing PPC (25, 29).

As evidenced by the risk assessment, another finding emerging from our review is the poor quality of data collection. Indeed, in the studies by Hall et al. and Davies et al. (17, 20), the efficacy of methadone was defined only as “improvement of pain,” without the use of dedicated scales. In the study by Mott et al. (18) when the patient was incapable of expressing pain, symptoms were evaluated by parents and clinicians as proxy measures. However, some of these symptoms, such as irritability and agitation, may be due to causes other than pain.

Madden et al. (10) used an *ad hoc*, non-validated pediatric symptoms assessment system. Only Palat et al. (19) used a standardized scale (NRS) but followed by poor data analysis. This may reflect a widespread difficulty to use dedicated scales for pain assessment in clinical practice, although several specific tools are available and validated events for children with communication issues or cognitive impairment (30). However, subjectively reported data such as those on pain should be interpreted with caution, since they can easily be altered by psychological and personal factors of the patient/caregiver (31, 32). Due to the small sample size and the qualitative nature for the majority of the observations, statistical comparison was not feasible. Other limitations of our review are that we searched only three databases and we did not include unpublished studies or unreported data.

Despite these methodological limitations of the retrieved studies, some interesting considerations can be raised on the role of methadone in the management of pain in PPC.

All identified studies documented the effectiveness of methadone in treating complex pain, either of nociceptive or neuropathic origin. Methadone was used both as first-line treatment and adjunctive therapy. The main reasons to initiate methadone were uncontrolled pain or adverse events on previous opioids.

This is in line with evidence from other fields, which suggests that methadone is a valuable option for nociceptive, mixed, and neuropathic pain, in this last case often as an adjuvant therapy (8, 10, 28).

While all studies agree on the indication of use, the data on how methadone was used are more difficult to interpret. Indeed, in some studies, such as Hall et al. methadone is evaluated in the treatment of pain at the EoL with consequently higher dosages and frequency of increase whereas in other studies, as for example Madden et al. the effectiveness of methadone as long-acting therapy was evaluated in advanced disease. Moreover, poor attention is paid to how methadone fits into patients' treatment plan, especially in the studies by Davies et al. and Madden et al. However, this limitation reflects a challenge in defining a unique standard for methadone use. This is a peculiarity of PPC, a setting in which the extreme variability of cases determines a need to adapt the use of drugs to the specific clinical situation. It could be interesting to better evaluate the different methadone requirement according to the stage of the diseases.

Moreover, methadone is available in different formulations, therefore allowing an administration tailored to the specific needs and desires of the patient (10, 20, 28).

No serious adverse events were reported in the identified studies, and no cases of cardiac arrhythmias—an adverse event potentially associated with methadone—were documented. However, their retrospective nature limits the reliability of the findings on methadone safety. This is particularly evident with respect to ECG monitoring, which was not performed in a standardized fashion; for example Davies et al. did not perform any ECG claiming that improving pain at the EoL outweighs this risk, whereas in Madden et al. who administered methadone in an earlier phase of disease, an ECG was performed in 81% of patients.

Based on limited data available in the pediatric population, QT elongation seems clinically irrelevant even in recipients of other drugs that may prolong QTc, with no case of torsade de pointes documented to date (10, 17, 18, 28). No routine ECG monitoring is recommended during methadone therapy, except for patients taking high doses of this molecule or at risk for QTc elongation (e.g., those with congenital cardiopathies, electrolytes imbalance or taking QTc prolonging drugs) (33, 34).

As reported by Mott et al. and Palat et al. (18, 19), common symptoms of the early phase of treatment with methadone include drowsiness, unsteadiness, itch, constipation, behavioral change, nausea, and tachycardia. At higher doses, methadone can also be associated with sedation, respiratory depression, neurotoxicity (myoclonus, hallucination) and hypoglycemia (8). Most deaths attributed to methadone overdose occur in the first 4–6 days of treatment due to its potential for accumulation (7, 23, 35).

Another concern about safety is potential interaction with other drugs that may induce or inhibit CYP enzymes (6, 10, 36), such as corticosteroids, fluconazole, cannabidiol, amitriptyline, carbamazepine, phenytoin (4, 10, 23). This risk should be carefully considered in PPC patients, who are poly-treated.

Although these concerns should be considered, identified studies seem to suggest the possibility of a safe administration of methadone also in the outpatient setting, with the possibility of a prolonged therapy (up to 307 days in the study by Palat et al. (8, 10, 19, 20). However, this option can be considered if parents are reliable and if there are resources to provide at least a specialized follow-up from remote.

The optimal dosing schedule of methadone is still under debate (37). In opioid-naive children, the studies identified in the present review suggest starting methadone at a dose of 0.05–0.1 mg/kg/day, from 2 to 4 times daily, followed by gradual titration (8, 17, 19). The strategy for optimal equianalgesic conversion from other opioids to methadone is more controversial. In adults, three main strategies of conversion to methadone are used in clinical practice (“rapid conversion”; “*ad libitum*”, and “3-days conversion”) (38), but they are not validated in pediatric patients so far. Available data in this population, although limited, a better outcome with rapid or gradual conversion (20), while the “as needed” approach could be more challenging especially in younger patients. Gradual conversion is considered safer compared with rapid conversion; with this approach, morphine dose is decreased by 30–50% per day while a loading dose of methadone (8, 25), calculated by the MEDD:TMDD conversion rate, is administered. In the studies analyzed, both Davies et al. and Mott et al. reviewed retrospectively equianalgesic conversion in PPC setting (18, 20); rapid conversion and gradual conversion were the most used strategies. Equianalgesic ratio (MEDD:TMDD) varied from 1:2 to 150:1, reflecting the high-individual variability of the pharmacokinetic of methadone.

Current recommendations suggest a 1:1 conversion rate in case of lower and brief prior exposure to opioids, and ratios of 20:1 or higher in case of greater and longer prior opioid exposure (8, 18, 20). Moreover, in patients on opioids but without adequate pain control, a rapid conversion and a starting dose of 0.1 mg/kg/dose would be reasonable (20). However, available evidence does not allow to recommend a general conversion table (8, 37), and therefore equianalgesic conversion has to be tailored to each single case, taking into account factors like the degree of opioid dose escalation required in previous weeks, route of administration, patient's weight, sedation score, mental status and respiratory rate (20). Therefore, and especially for patients on long-lasting prior treatment with high-dose opioids, methadone conversion should be proposed by an experienced physician within hospital monitoring or in the inpatient setting (35, 39, 40).

## Conclusion

Available evidence, although collected in a limited number of patients and with an overall modest methodological quality, seems to suggest that methadone could represent a suitable strategy to treat complex pain in the PPC setting. It is however important to point out that no firm recommendation for its use can be made. Health care providers should be adequately trained before the use of methadone and careful monitoring of this treatment during the entire therapy course is crucial.

To date, there is a large gap in the literature regarding the use of methadone in children with life-limited and life-threatening non-cancer conditions. The future goal will be to develop methodologically well-conducted interventional, if faisable, or observational studies with larger numbers that can lead to strong recommendations for the use of this drug, which, in the few studies conducted to date, has been shown to hold the potential for efficacy and safety for use in this population.

Areas for further study on the use of methadone in pediatrics concern its use in intensive care and its use in the treatment of pain in oncology. However, most of the available literature concerns the adult population. Further evidence in PPC is needed for the correct use of methadone in pain management.

On these bases, we believe that further research on methadone in the PPC setting is warranted. Randomized controlled trials may not be feasible, but high-quality prospective studies on the use of methadone in PPC should be planned and conducted.

## Author Contributions

FBene, SZ, and AS accomplished the bibliographic review, selection and reading of articles, and drafted the manuscript. IA has revised the search strategy, resolved any disagreements, and reviewed the manuscript. FBeni supervised the entire research work and the writing of the article. All authors contributed to the article and approved the submitted version.

## Conflict of Interest

The authors declare that the research was conducted in the absence of any commercial or financial relationships that could be construed as a potential conflict of interest.

## Publisher's Note

All claims expressed in this article are solely those of the authors and do not necessarily represent those of their affiliated organizations, or those of the publisher, the editors and the reviewers. Any product that may be evaluated in this article, or claim that may be made by its manufacturer, is not guaranteed or endorsed by the publisher.

## References

[1] Organization GWH. Integrating Palliative Care and Symptom Relief Into Paediatrics: A WHO Guide for Health Care Planners, Implementers and Managers. Geneva.

[2] Friedrichsdorf SJ Postier AC Andrews GS Hamre KE Steele R Siden H. Pain reporting and analgesia management in 270 children with a progressive neurologic, metabolic or chromosomally based condition with impairment of the central nervous system: cross-sectional, baseline results from an observational, longitudinal study. J Pain Res. (2017) 10:1841–52. 10.2147/JPR.S138153 28831272 PMC5548270

[3] Beecham E Candy B Howard R McCulloch R Laddie J Rees H. Pharmacological interventions for pain in children and adolescents with life-limiting conditions. Cochrane Database Syst Rev. (2015) 2015:Cd010750. 10.1002/14651858.CD010750.pub2 25768935 PMC6481584

[4] Anghelescu DL Snaman JM Trujillo L Sykes AD Yuan Y Baker JN. Patient-controlled analgesia at the end of life at a pediatric oncology institution. Pediatr Blood Cancer. (2015) 62:1237–44. 10.1002/pbc.25493 25820345 PMC4433603

[5] Mherekumombe MF Collins JJ. Patient-controlled analgesia for children at home. J Pain Symptom Manage. (2015) 49:923–7. 10.1016/j.jpainsymman.2014.10.007 25546288

[6] Fenton C Lyseng-Williamson KA. Methadone is an option to treat cancer pain in carefully selected patients. Drugs Ther Perspect. (2020) 36:502–8. 10.1007/s40267-020-00772-4

[7] Edmonds KP Saunders IM Willeford A Ajayi TA Atayee RS. Emerging challenges to the safe and effective use of methadone for cancer-related pain in paediatric and adult patient populations. Drugs. (2020) 80:115–30. 10.1007/s40265-019-01234-6 31820362

[8] Friedrichsdorf SJ. From tramadol to methadone: opioids in the treatment of pain and dyspnea in pediatric palliative care. Clin J Pain. (2019) 35:501–8. 10.1097/AJP.0000000000000704 30985399

[9] Thigpen JC Odle BL Harirforoosh S. Opioids: a review of pharmacokinetics and pharmacodynamics in neonates, infants, and children. Eur J Drug Metab Pharmacokinet. (2019) 44:591–609. 10.1007/s13318-019-00552-0 31006834

[10] Madden K Mills S Dibaj S Williams JL Liu D Bruera E. Methadone as the initial long-acting opioid in children with advanced cancer. J Palliat Med. (2018) 21:1317–21. 10.1089/jpm.2017.0712 29664690

[11] Womer J Zhong W Kraemer FW Maxwell LG Ely EA Faerber JA. Variation of opioid use in pediatric inpatients across hospitals in the U.S. J Pain Symptom Manage. (2014) 48:903–14. 10.1016/j.jpainsymman.2013.12.241 24703942

[12] Norris S Minkowitz S Scharbach K. Pediatric palliative care. Prim Care. (2019) 46:461–73. 10.1016/j.pop.2019.05.010 31375193

[13] Emerson ND Bursch B. Ethical issues in providing end-of-life cancer care for transitional-aged youth. J Clin Psychol Med Settings. (2021) 28:781–8. 10.1007/s10880-021-09764-6 33598787

[14] Craig F Abu-Saad Huijer H Benini F Kuttner L Wood C Feraris PC. [IMPaCCT: standards of paediatric palliative care]. Schmerz. (2008) 22:401–8. 10.1007/s00482-008-0690-4 18516628

[15] Liberati A Altman DG Tetzlaff J Mulrow C Gøtzsche PC Ioannidis JPA. The PRISMA statement for reporting systematic reviews and meta-analyses of studies that evaluate healthcare interventions: explanation and elaboration. BMJ. (2009) 339:b2700. 10.1136/bmj.b2700 19622552 PMC2714672

[16] Munn Z Barker TH Moola S Tufanaru C Stern C McArthur A. Methodological quality of case series studies: an introduction to the JBI critical appraisal tool. JBI Evid Synth. (2020) 18:2127–33. 10.11124/JBISRIR-D-19-00099 33038125

[17] Hall EA Sauer HE Habashy C Anghelescu DL. Methadone for cancer pain in pediatric end-of-life care. Am J Hosp Palliat Care. (2021) 38:914–9. 10.1177/1049909120963641 33000633

[18] Mott C Sarpal A Moss K Herbert A. Methadone for analgesia in children with life-limiting illness: experience from a tertiary children's health service. Children. (2018) 5:86. 10.3390/children5070086 29954057 PMC6068960

[19] Palat G Algotsson C Rayala S Haridass V Nethagani J Rapelli V. The use of methadone in pediatric cancer pain—a retrospective study from a Governmental Cancer Center in India. Indian J Palliat Care. (2021) 27:133–8. 10.4103/IJPC.IJPC_109_20 34035631 PMC8121219

[20] Davies D DeVlaming D Haines C. Methadone analgesia for children with advanced cancer. Pediatr Blood Cancer. (2008) 51:393–7. 10.1002/pbc.21584 18428431

[21] Barbosa Neto JO Garcia MA Garcia JBS. Revisiting methadone: pharmacokinetics, pharmacodynamics and clinical indication. Revista Dor. (2015) 16:60–6. 10.5935/1806-0013.20150012

[22] Toombs JD Kral LA. Methadone treatment for pain states. Am Fam Physician. (2005) 71:1353–8. 15832538

[23] Chhabra S Bull J. Methadone. Am J Hosp Palliat Care. (2008) 25:146–50. 10.1177/1049909107312597 18445864

[24] Reach JS Yue JJ Narayan D Kaye AD Vadivelu N. Perioperative Pain Management for Orthopedic and Spine Surgery. Oxford: Oxford University Press (2018). 10.1093/med/9780190626761.001.0001

[25] Goldman A Hain R Liben S. Oxford Textbook of Palliative Care for Children. Oxford: OUP Oxford (2012). 10.1093/med/9780199595105.001.0001

[26] Cooper TE Fisher E Gray AL Krane E Sethna N van Tilburg MA. Opioids for chronic non-cancer pain in children and adolescents. Cochrane Database Syst Rev. (2017) 7:Cd012538. 10.1002/14651858.CD012538 28745394 PMC6477875

[27] Wiffen PJ Cooper TE Anderson AK Gray AL Grégoire MC Ljungman G. Opioids for cancer-related pain in children and adolescents. Cochrane Database Syst Rev. (2017) 7:Cd012564. 10.1002/14651858.CD012564 28722116 PMC6484393

[28] Habashy C Springer E Hall EA Anghelescu DL. Methadone for pain management in children with cancer. Paediatr Drugs. (2018) 20:409–16. 10.1007/s40272-018-0304-2 30047027

[29] Benini F Pappadatou D Bernadá M Craig F De Zen L Downing J. International standards for pediatric palliative care: from IMPaCCT to GO-PPaCS. J Pain Symptom Manage. (2022) 63:e529–e543. 10.1016/j.jpainsymman.2021.12.031 35031506

[30] Hauer J Houtrow AJ. Pain assessment and treatment in children with significant impairment of the central nervous system. Pediatrics. (2017) 139:e20171002. 10.1542/peds.2017-1002 28562301

[31] Jayanath S Ong LC Marret MJ Fauzi AA. Parent-reported pain in non-verbal children and adolescents with cerebral palsy. Dev Med Child Neurol. (2016) 58:395–401. 10.1111/dmcn.12943 26510627

[32] Lifland BE Mangione-Smith R Palermo TM Rabbitts JA. Agreement between parent proxy report and child self-report of pain intensity and health-related quality of life after surgery. Acad Pediatr. (2018) 18:376–83. 10.1016/j.acap.2017.12.001 29229566 PMC5936667

[33] Chou R Cruciani RA Fiellin DA Compton P Farrar JT Haigney MC. Methadone safety: a clinical practice guideline from the American Pain Society and College on problems of drug dependence, in collaboration with the Heart Rhythm Society. J Pain. (2014) 15:321–37. 10.1016/j.jpain.2014.01.494 24685458

[34] Krantz MJ Martin J Stimmel B Mehta D Haigney MC. QTc interval screening in methadone treatment. Ann Intern Med. (2009) 150:387–95. 10.7326/0003-4819-150-6-200903170-00103 19153406

[35] World Health Organization. Linee guida dell'OMS sul trattamento farmacologico del dolore persistente nei bambini con patologie croniche gravi (2014). Traduzione Italiana del Document “WHO Guidelines on the Pharmacological Treatment of Persisting Pain in Children with Medical Illnesses. Geneva: World Health Organization (2012). 23720867

[36] Rasmussen VF Lundberg V Jespersen TW Hasle H. Extreme doses of intravenous methadone for severe pain in two children with cancer. Pediatr Blood Cancer. (2015) 62:1087–90. 10.1002/pbc.25392 25641929

[37] Fife A Postier A Flood A Friedrichsdorf SJ. Methadone conversion in infants and children: retrospective cohort study of 199 pediatric inpatients. J Opioid Manag. (2016) 12:123–30. 10.5055/jom.2016.0324 27194197

[38] McLean S Twomey F. Methods of rotation from another strong opioid to methadone for the management of cancer pain: a systematic review of the available evidence. J Pain Symptom Manage. (2015) 50:248–59.e1. 10.1016/j.jpainsymman.2015.02.029 25896106

[39] Zernikow B Michel E Craig F Anderson BJ. Pediatric palliative care: use of opioids for the management of pain. Paediatr Drugs. (2009) 11:129–51. 10.2165/00148581-200911020-00004 19301934

[40] Friedrichsdorf SJ Kang TI. The management of pain in children with life-limiting illnesses. Pediatr Clin North Am. (2007) 54:645–72. 10.1016/j.pcl.2007.07.007 17933616

